# Finite element analysis of optimized novel additively manufactured non-articulating prostheses for cervical total disc replacement

**DOI:** 10.3389/fbioe.2023.1182265

**Published:** 2023-06-01

**Authors:** Ming-Kai Hsieh, Ching-Lung Tai, Yun-Da Li, De-Mei Lee, Cheng-Yi Lin, Tsung-Ting Tsai, Po-Liang Lai, Weng-Pin Chen

**Affiliations:** ^1^ Department of Orthopaedic Surgery, Spine Section, Bone and Joint Research Center, Chang Gung Memorial Hospital and Chang Gung University College of Medicine, Taoyuan, Taiwan; ^2^ Department of Biomedical Engineering, Chang Gung University, Taoyuan, Taiwan; ^3^ Department of Mechanical Engineering, Chang Gung University, Taoyuan, Taiwan; ^4^ Department of Mechanical Engineering, National Taipei University of Technology, Taipei, Taiwan

**Keywords:** biomechanics, hybrid artificial cervical disc, additive manufacture technology, polycarbonate urethane, finite element analysis, lattice structure

## Abstract

Ball-and-socket designs of cervical total disc replacement (TDR) have been popular in recent years despite the disadvantages of polyethylene wear, heterotrophic ossification, increased facet contact force, and implant subsidence. In this study, a non-articulating, additively manufactured hybrid TDR with an ultra-high molecular weight polyethylene core and polycarbonate urethane (PCU) fiber jacket, was designed to mimic the motion of normal discs. A finite element (FE) study was conducted to optimize the lattice structure and assess the biomechanical performance of this new generation TDR with an intact disc and a commercial ball-and-socket Baguera^®^C TDR (Spineart SA, Geneva, Switzerland) on an intact C5-6 cervical spinal model. The lattice structure of the PCU fiber was constructed using the Tesseract or the Cross structures from the IntraLattice model in the Rhino software (McNeel North America, Seattle, WA) to create the hybrid I and hybrid II groups, respectively. The circumferential area of the PCU fiber was divided into three regions (anterior, lateral and posterior), and the cellular structures were adjusted. Optimal cellular distributions and structures were A2L5P2 in the hybrid I and A2L7P3 in the hybrid II groups. All but one of the maximum von Mises stresses were within the yield strength of the PCU material. The range of motions, facet joint stress, C6 vertebral superior endplate stress and path of instantaneous center of rotation of the hybrid I and II groups were closer to those of the intact group than those of the Baguera^®^C group under 100 N follower load and pure moment of 1.5 Nm in four different planar motions. Restoration of normal cervical spinal kinematics and prevention of implant subsidence could be observed from the FE analysis results. Superior stress distribution in the PCU fiber and core in the hybrid II group revealed that the Cross lattice structure of a PCU fiber jacket could be a choice for a next-generation TDR. This promising outcome suggests the feasibility of implanting an additively manufactured multi-material artificial disc that allows for better physiological motion than the current ball-and-socket design.

## Introduction

Total disc replacement (TDR) has been proven to preserve level motion at the treated level and reduce adjacent segment degeneration, which are advantages over anterior cervical discectomy and fusion (ACDF) (Findlay et al., 2018). Between 2006 and 2013, a total of 1,059,403 ACDF and 13,099 TDR surgeries were performed in the United States. During this same time period, the annual number of ACDF and TDR increased non-linearly by 5.7% and 190% (Saifi et al., 2018). The preservation of physiologic motion at the treated level leads to longevity of the facet joints and decreases the adjacent segment degeneration rate, which would otherwise lead to additional revision surgery (Bhattacharya et al., 2019; Rajakumar et al., 2017; Yang et al., 2017). Ball-and-socket TDR have been more popular than other designs in recent years, but their potential disadvantages include polyethylene (PE) wear, heterotrophic ossification, increased contact forces, and disc subsidence and migration (Virk et al., 2021; Shen et al., 2021; Shen et al., 2022; Cao et al., 1976; Parish et al., 2020). A new-generation prosthesis with a compressible central core allows for six kinematic degrees of freedom and mimics normal biomechanics has been developed and used (Patwardhan et al., 2012; Phillips et al., 2021; Oltulu et al., 2019). The non-articulating TDR was designed to model normal discs by using PE fiber as the annulus, polycarbonate urethane (PCU) core as the nucleus pulposus, and covered polymer to catch soft tissue growth and debris. However, the overall biomechanical performance of the fiber network of the TDR was determined not only by the geometric properties of the layered material but also by the physical similarity, complexity, lattice geometry, and distributions of the materials, which should be taken into consideration (Mahbod and Asgari, 2019; Maconachie et al., 2019). The open porous cellular architecture of the lattice plays an important role in energy absorption and stress distribution (Mahbod and Asgari, 2019; Maconachie et al., 2019; Gutiérrez, 2020).

The applications of additive manufacturing (AM) technologies, also known as rapid prototyping or three-dimensional (3-D) printing, in the biomedical field have increased substantially in recent years (Puppi and Chiellini, 2020; Kumar et al., 2021). Five key benefits that AM has over traditional manufacturing include cost, speed, quality, innovation/transformation, and impact. Small volume, on-demand and component manufacturing enables quality improvement and time and cost savings (Attaran, 2017). AM 3D printing has been used in several biomedical applications, including anatomical models, customized implants, and tissue and organ fabrication (Attaran, 2017; Mobbs et al., 2017). Preoperative planning in the reconstruction of C1-2 chordoma, sacral osteosarcoma, and vertebral fracture was also successfully applied (Siu et al., 2018; Kim et al., 2017; Phan et al., 2016).

With recent progress in micro-fabrication technology by AM, lattice structures can now be made with diameters ranging from millimeter to submicron levels (Tancogne-Dejean et al., 2016). The mechanical and biological performance of implants can be greatly affected by internal architecture, and implant design can be tailored by using AM techniques.

Our study utilized additive manufacturing to create a TDR with a multi-material elastomeric design consisting of a PE core and a PCU fiber jacket. This design was chosen to replicate the anisotropic mechanical properties of natural discs at the frequently degenerated C5-6 cervical level (Teraguchi et al., 2014). The purpose of our finite element (FE) study was to optimize the cellular structure, density and distribution of the PCU fibers in the TDR produced by AM. The core stress, C6 superior endplate stress, facet joint force, and range of motion at the treated level of the cervical spine were compared between the two optimized AM TDR and a commercial product (Baguera^®^C, Spineart SA, Geneva, Switzerland). Biomechanical comparison and finite element analysis of various commercially available cervical artificial discs have been conducted in recent years (Kowalczyk et al., 2011; Lin et al., 1976; Chen et al., 2018; Choi et al., 2020). To the best of the author’s knowledge, this is the first finite element study optimizing an additively manufactured hybrid TDR and comparing them with a commercially available TDR with ball-and-socket designs.

## Materials and methods

### Development of an FE C5-6 intact cervical spine model

An FE model of a C5-C6 spine segment was developed from computed tomography (CT) images of the cervical spine of a 55 years/o male subject (The Visible Human Project, National Library of Medicine, Bethesda, MD, United States) at 1-mm intervals with an original disc height of 5.5 mm as the baseline model (Kamal and Rouhi, 2016a). The development process included the following steps: (1) The 3-D reconstruction of the solid volume based on the CT images was set up by using commercial segmentation and visualization software (Amira 4.1, TGS, San Diego, CA). (2) The solid model was then exported into a readable input file for the SolidWorks 2014 CAD software (Solidworks Corp., Boston, MA, United States), where the intervertebral disc was created. (3) The bony components and intervertebral disc were meshed using linear tetrahedral elements by FE pre-processing software (HyperMesh 2017, Altair Engineering, Inc., Troy, MI, United States). The thickness of the cortex and endplate were set as 0.5 mm (Kamal and Rouhi, 2016a; Kwon et al., 2020; Liu et al., 2020). (4) The material properties and loading/boundary conditions of the FE analysis were set up, and nonlinear, quasi-static analysis was performed using Abaqus 2018/CAE software (Simulia Corp., Prvidence, RI, United States).

The detailed material parameters used for the parts of the C5-C6 spine model, including cortical bone, cancellous bone, posterior bony elements, endplates, nucleus pulposus, annulus fibrosus, and annulus ground substance, are shown in Table 1. All materials were assumed to be linear elastic, homogeneous and isotropic, except orthotropic in cancellous bone and hyperelastic in nucleus pulposus and annulus ground substance (Kowalczyk et al., 2011; Lin et al., 1976; Chen et al., 2018; Choi et al., 2020).

**TABLE 1:** Material parameters used for the C5-C6 spine model.

	Young’s modulus (MPa)	Poisson’s ratio (ν)	Element type	References
Cortical Bone	10,000	0.3	C3D4	Mackiewicz et al. (2016)
Cancellous Bone	E_xx_ = 100	v_xy_ = 0.3 v_xz_ = 0.1	C3D4	Mackiewicz et al., 2016, Whyne et al., 2001
	E_yy_ = 100			
	E_zz_ = 300			
	G_xy_ = 38			
	G_yz_ = 77			
	G_zx_ = 77			
Posterior Bone	3,500	0.3	C3D4	Toosizadeh and Haghpanahi (2011)
Endplate	500	0.4	C3D4	Zhang et al. (2006)
Annulus Ground Substance (Mooney–Rivlin)	C_10_ = 0.56	0.45	C3D4	Toosizadeh and Haghpanahi, (2011), SchmidtHeuer et al., 2006
	C_01_ = 0.14			
Annulus	175	0.3	SFM3D4	
Fibrosus				
Nucleus	C_10_ = 0.12	0.4999	C3D4	Mackiewicz et al. (2016)
Pulposus (Mooney–Rivlin)	C_01_ = 0.09			
Rigid Body	10^12^	0.3	C3D4	Toosizadeh and Haghpanahi, (2011), SchmidtHeuer et al., 2006

Six major groups of ligaments of the C5-C6 spine model, including anterior longitudinal (ALL), posterior longitudinal (PLL), ligament flavum (LF), facet capsular (FCL), interspinous (ISL), and supraspinous (SSL), were created. The points of attachment of the ALL and PLL were along the anterior and posterior surfaces of the vertebral bodies. The FCL was attached between the articular processes, the LF between the lower margin of the C5 laminae to the upper-third of the C6 laminae, and the ISL between the two spinous processes. The ligaments were modeled using two-node wire nonlinear link elements that permitted only nonlinear tensile force transmission and are listed in Table 2 (Wheeldon et al., 2008).

**TABLE 2:** Force-displacement data used for the ligaments in the spine model (Wheeldon et al., 2008).

All		PLL		ISL, SSL		LF		FCL	
Displacement (mm)	Force (N)	Displacement (mm)	Force (N)	Displacement (mm)	Force (N)	Displacement (mm)	Force (N)	Displacement (mm)	Force (N)
0	0	0	0	0	0	0	0	0	0
1	35.5	0.9	1.33	1.2	0.75	1.7	2.2	1.7	2.452
2	64.9	2	29.0	2.7	16.9	3.74	45.9	3.9	53.6
4	89.7	3	51.4	4.0	24.4	5.6	82.9	5.8	87.9
5	108.6	4	71.38	5.4	29.5	7.48	119.6	7.7	109.4
6	119.6	5	85.8	6.7	32.9	9.35	133.7	9.7	125.8
-	-	6	94.7	8.1	34.9	11.3	147.2	11.5	134.8

### Development and optimization of the AM hybrid TDRs

The overall design process of the additive manufacturing of hybrid TDRs consisted of four steps.

**FIGURE 1:**
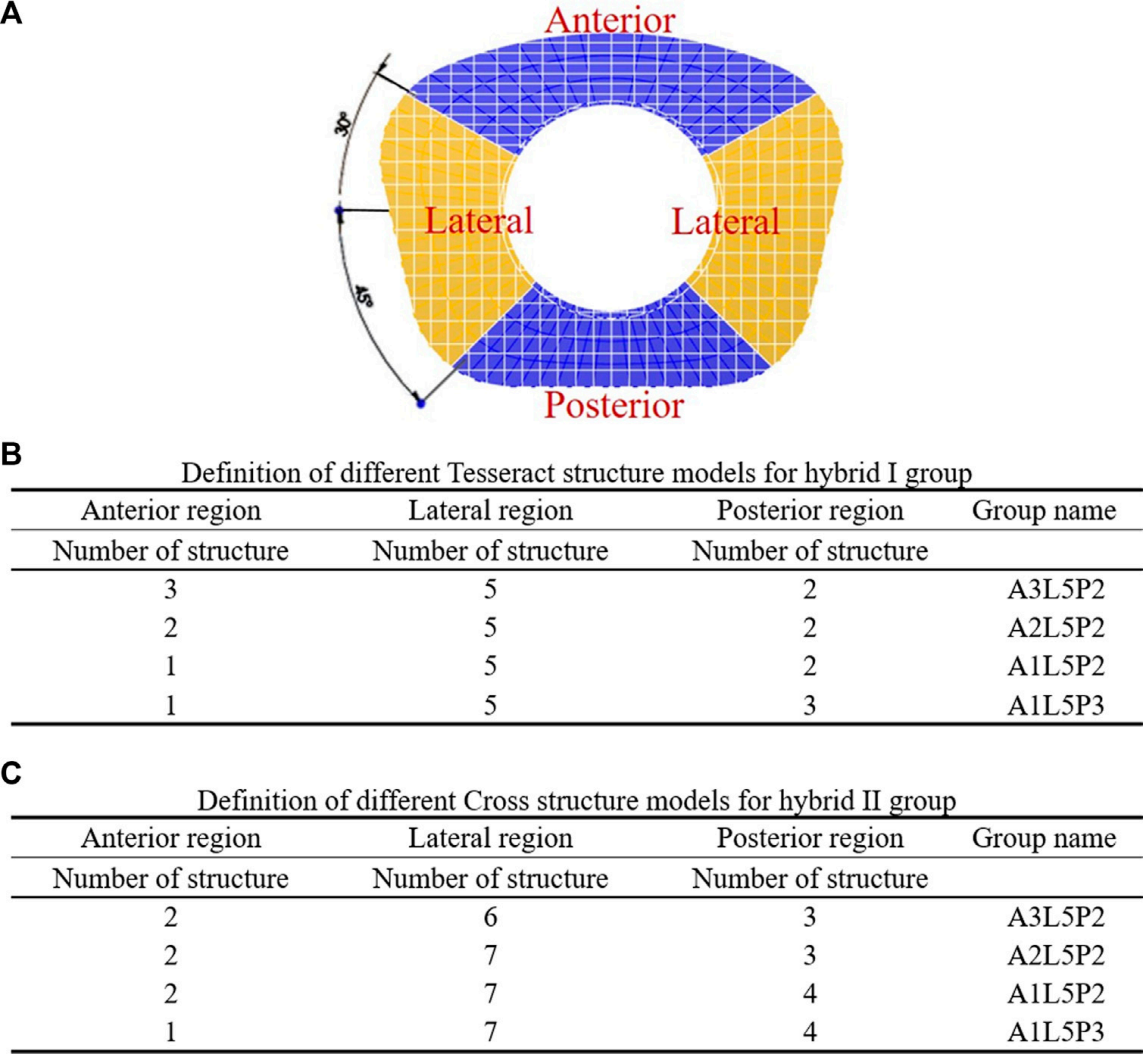
Division of circumferential regions of the artificial fiber jacket structure **(A)**. Definition of different Tesseract structure models for the hybrid I group **(B)** and Cross structure models for the hybrid II group **(C)**.

First, the referenced geometric parameters were input. The additively manufactured TDR, a multi-material elastomeric design with ultra-high molecular weight PE (UHMWPE) core and PCU fiber jacket, was designed. The superior and inferior plates of the AM TDR were constructed with stainless steel (316L). The geometric data of the UHMWPE core were 5.8 mm in height, 14.0 mm in anterior-posterior length, and 17.9 mm in width, and the distance between the core and the superior plate was 0.08 mm (Moroney et al., 1988). The lattice structure of the PCU fiber jacket was constructed using the Tesseract or the Cross structures from the IntraLattice model in the Rhinoceros 6 software (Rhino, McNeel North America, Seattle, WA) to create the Hybrid I and Hybrid II groups, respectively (Figure 1). The circumferential area of the PCU fiber jacket was divided into three different regions (Figure 1A), and the lattice structures were adjusted into different models: A3L5P2, A2L5P2, A1L5P2 and A1L5P3 in the Hybrid I group (Figure 1B) and A2L6P3, A2L7P3, A2L7P4 and A1L7P4 in the Hybrid II group (Figure 1C). According to the biomechanical performance in the range of motion (ROM) under various loadings compared to the intact model, the optimal lattice structures from the Hybrid I and Hybrid II groups were chosen, and their biomechanical performances were then compared with a commercially available Baguera^®^C cervical joint prosthesis (Spineart SA, Geneva, Switzerland).

### Development of the FE model of the Baguera^®^C TDR

**FIGURE 2:**
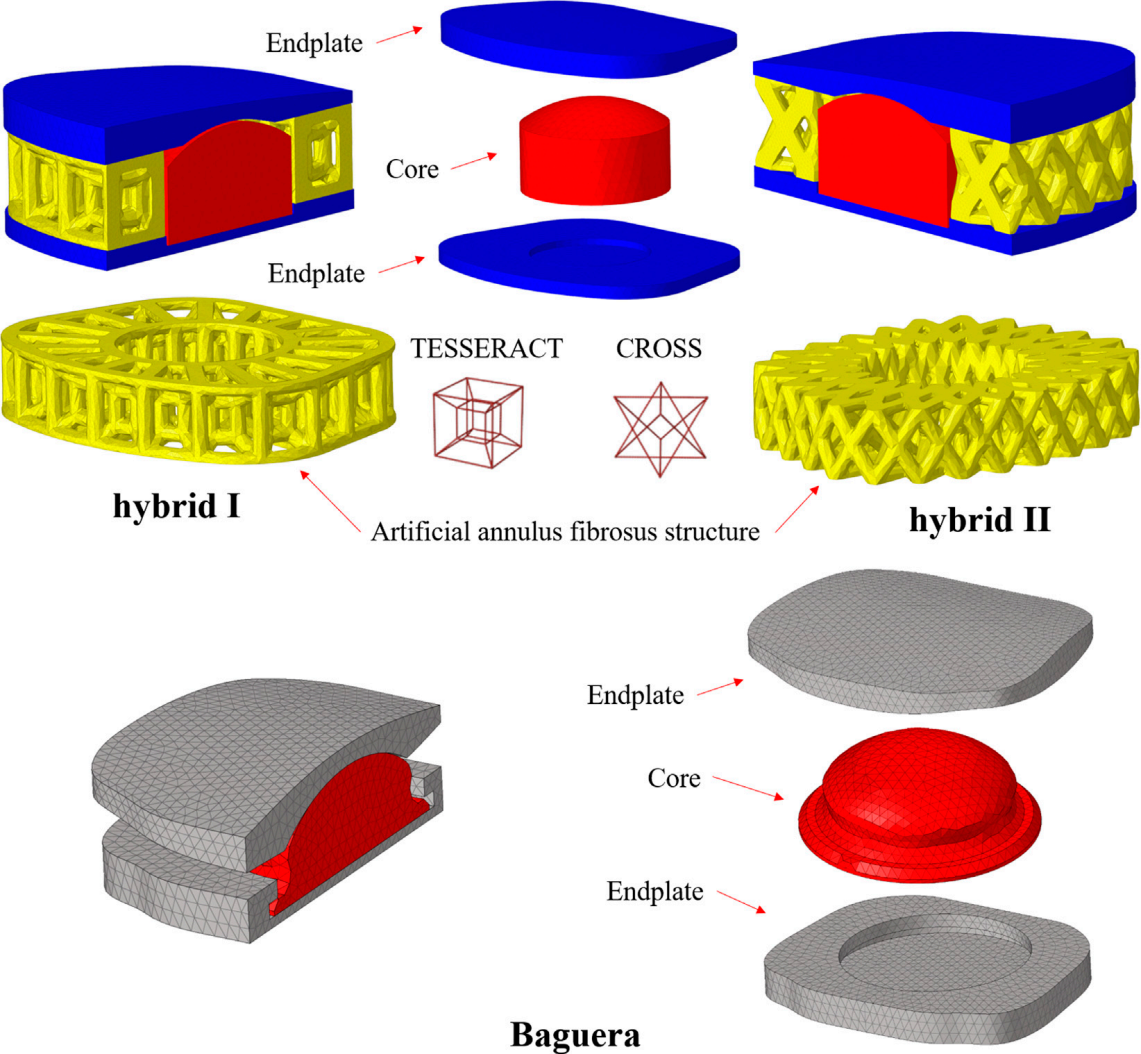
Detailed parts of the hybrid I, hybrid II and Baguera^®^C TDR models.

The 3D computer-aided design (CAD) model of the Baguera^®^C TDR was reconstructed through an ATOS scanning system (GOM mbH, Braunschweig, Germany) with dimensions of 13, 16, and 7 mm in length, width and height, respectively (Figure 2). The internal nucleus of the Baguera group is able to translate ±0.3 mm, anterior-posteriorly and laterally. Two degrees of axial rotation of the nucleus and 8 degrees of sagittal and coronal rotation of the entire implant were set before the analysis. The CAD model was exported into a readable input file for Solidworks 2014 software (Solidworks Corp., Boston, MA, United States), and then a finite element model was created with tetrahedral elements in Hypermesh 2017 FE pre-processing software (HyperMesh, Altair Engineering, Inc., Troy, MI). The material properties and loading/boundary conditions were set up, and FE analysis was performed with Abaqus 2018/CAE software (Simulia Corp., Prvidence, RI, United States).

### Material parameters setup for TDR finite element models

**FIGURE 3:**
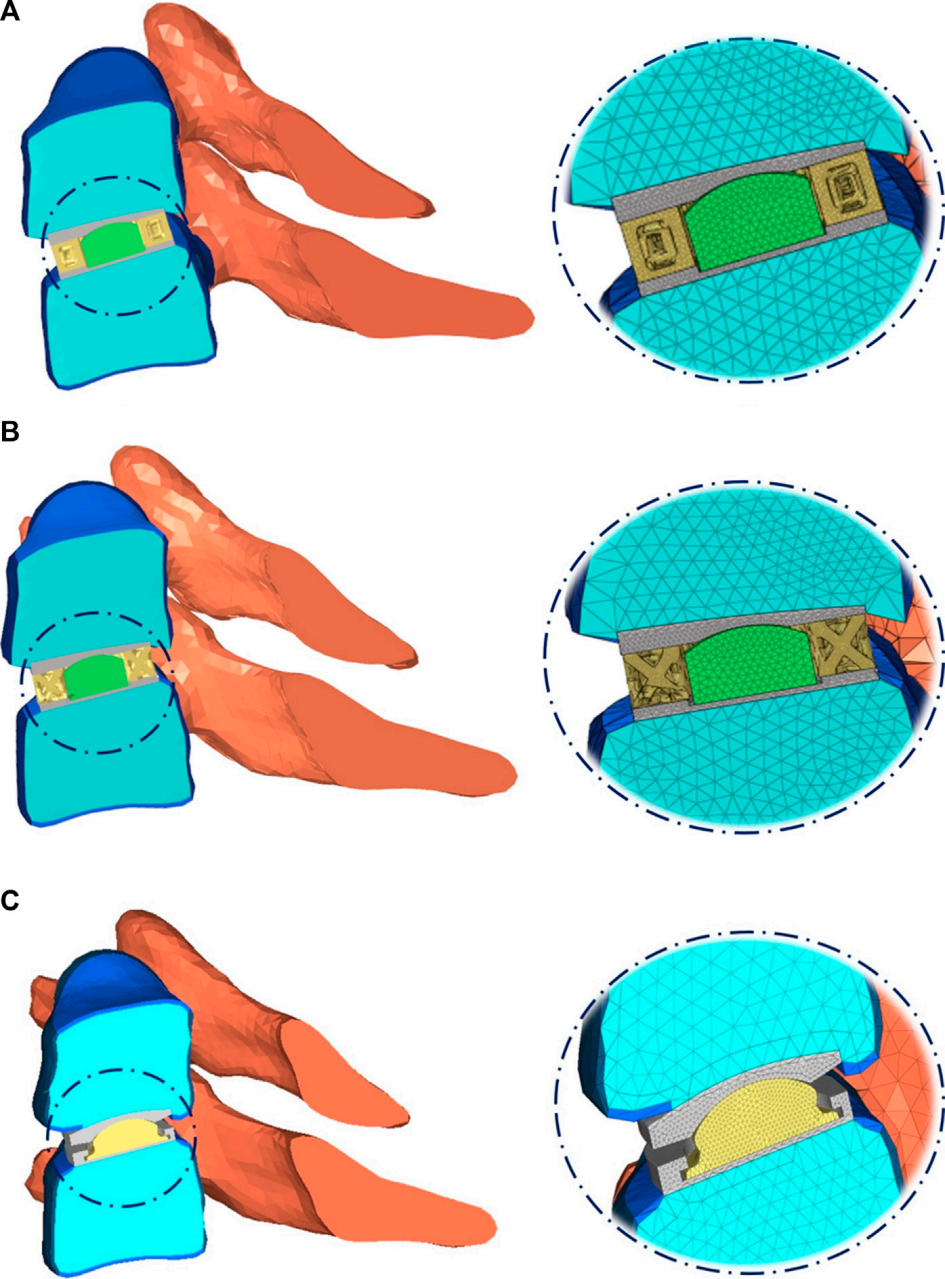
Schematic diagram of three TDR implantation models: **(A)** hybrid I group, **(B)** hybrid II group, **(C)** Baguera^®^C group.

The detailed parts of the Hybrid I and Hybrid II models included endplates, a fiber jacket, and a core and the Baguera^®^C model included an endplate and a core. The material parameters were set up and are listed in Table 3. All materials were assumed to be linear elastic and homogeneous. The schematic diagrams for the three TDR implantation models are shown in Figure 3.

**TABLE 3:** Material parameters used for the artificial disc materials (Kamal and Rouhi, 2016b), (Jacobs et al., 2017).

	Young’s modulus (MPa)	Poisson’s ratio (ν)	Element type	References
Ti-6-Al-4-V	110,000	0.3	C3D4	Kamal and Rouhi (2016b)
316 L	52,000	0.3	C3D4	
Stainless Steel				
PCU 75D	188	0.3	C3D4	Jacobs et al. (2017)
UHMWPE	800	0.3	C3D4	Kamal and Rouhi (2016b)

### Loading and boundary conditions

**FIGURE 4:**
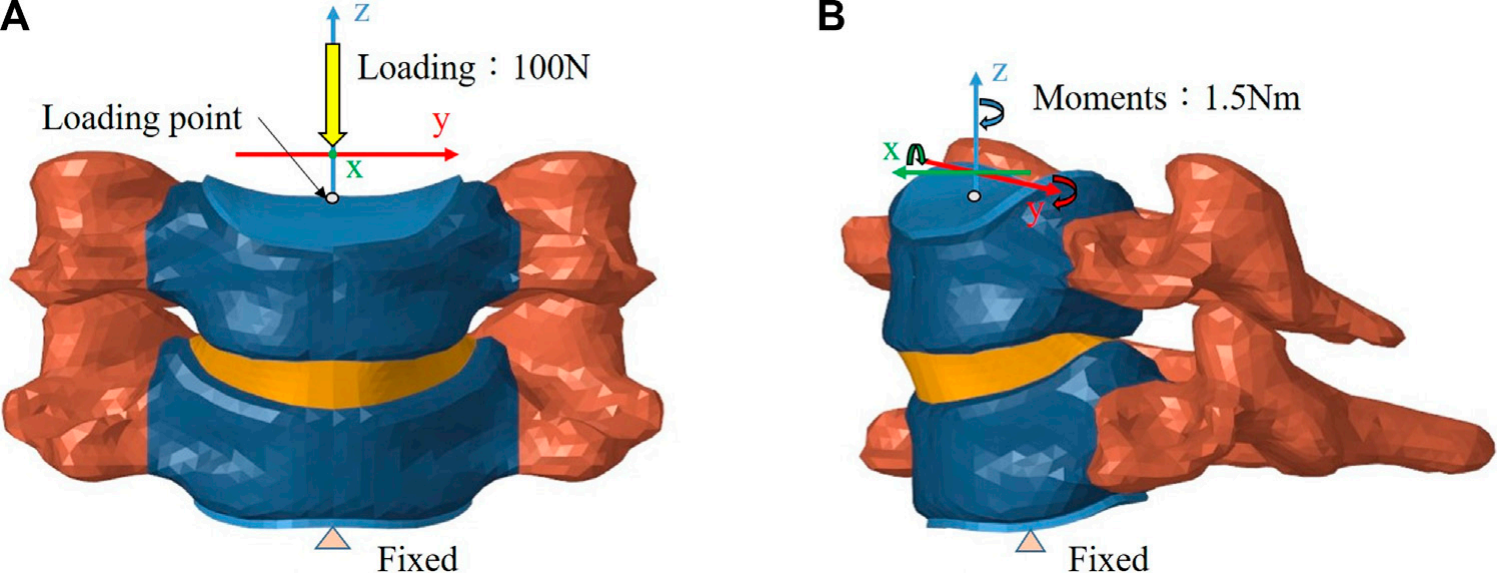
Finite element model of the intact C5–C6 motion segment with loading/boundary conditions: **(A)** coronal view, **(B)** sagittal view.

The inferior surface of the C6 vertebra was fixed in all degrees of freedom, while a follower load of 100 N that simulating the head weight and local muscle motion during daily activity was applied on the superior surface of the C5 vertebra (Bell et al., 2018; Finn et al., 2009). In loading control, pure moments of 1.5 Nm were generated by a force couple during fiexion, extension, lateral bending, and axial rotation of the cervical spine respectively (Figure 4) (Bell et al., 2018; Finn et al., 2009). A friction coefficient of 0.1 was established for the ball and socket joints of the implants (Galbusera et al., 2008). The boundary condition between the implant endplates and vertebral bodies was set to merge to achieve fully restrained components. To prevent any relative sliding, a Tie contact was implemented between the artificial annulus fibrosus and implant endplates.

### Finite element model convergence test

The convergence criteria for the FE models were selected as the total strain energy (ALLSE in Abaqus 2018/CAE software) between different mesh refinements to be less than 5%. The final analysis mesh sizes were chosen to be 1.3 mm for the intact group, 0.2 mm for the Hybrid I group, 0.15 mm for the Hybrid II group and 0.2 mm for the Baguera group after the mesh convergence tests (Supplementary Table S1).

### Biomechanical evaluation

To compare the primary stabilizing properties of the three TDR groups, the ROMs of the operated C5-C6 motion segment with the Hybrid I, Hybrid II, and Baguera groups were compared to those of the Intact group under loadings of 100 N follower load and pure moment of 1.5 Nm in four different motions (flexion, extension, lateral bending, and axial rotation). To evaluate the strength of the TDRs and the tendency of prosthesis subsidence, the stress distributions of the core, facet joint and C6 vertebral superior endplate were analyzed for different motions. The quality of the spine movement was evaluated by the instantaneous center of rotation (ICR). The detailed ICR paths were calculated from the relative positions of the vertebrae during motion as described in the literature (Muhlbauer et al., 2020).

## Results

### Model validation

**FIGURE 5:**
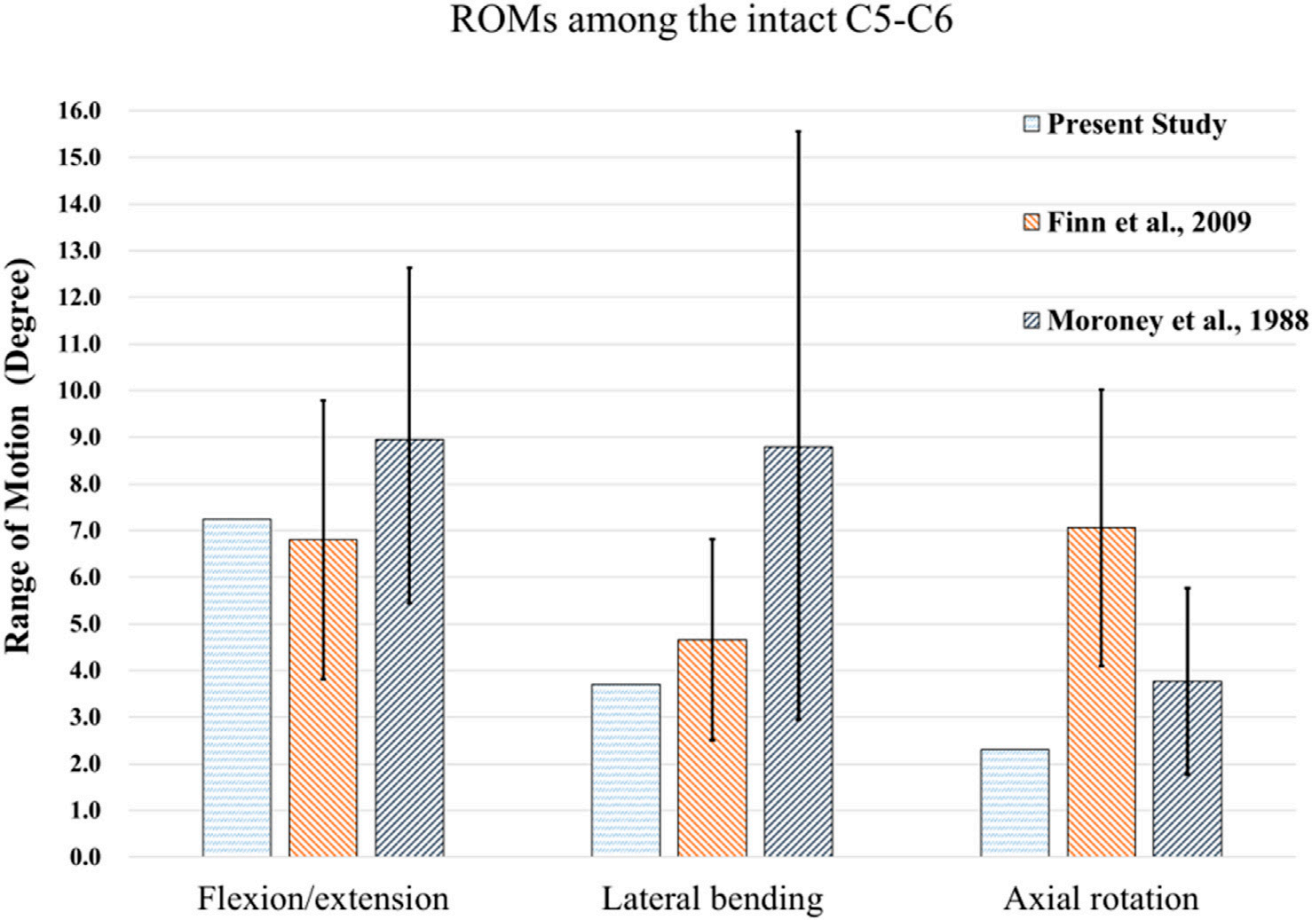
Comparisons of ROMs for the intact C5-C6 model and those obtained from previous biomechanical studies (Moroney et al., 1988), (Finn et al., 2009).

Figure 5 shows the comparison of ROMs for the intact C5-C6 model and those obtained in previous biomechanical studies for moments of 1.5 Nm (Finn et al., 2009; Purushothaman et al., 2020). The ROMs of the intact C5-C6 FE model were 7.25, 3.7 and 2.31° during flexion/extension, lateral bending and axial rotation, respectively. All ROMs of the intact model were comparable with previously obtained experimental data, and the comparison successfully verified the intact model used in the current study for further biomechanical analysis of implantation groups.

### Optimization of two AM TDR groups

**FIGURE 6:**
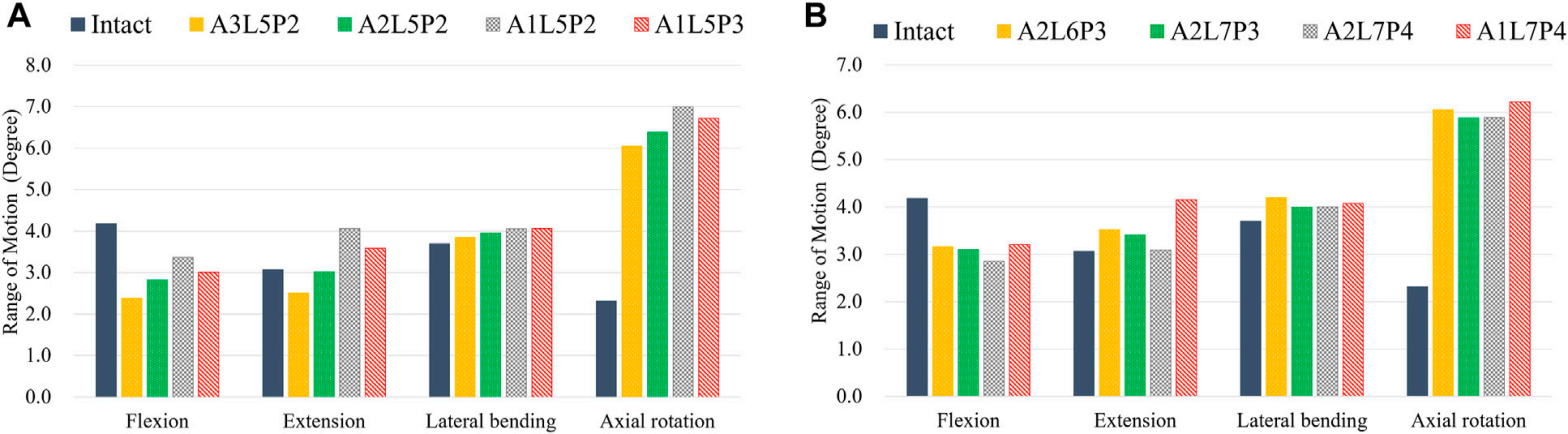
ROMs for different lattice structure models for the hybrid I and hybrid II groups for different motions (FL: flexion, EX: extension, LB: lateral bending, and AR: axial rotation) compared to those of the intact group: **(A)** hybrid I group, **(B)** hybrid II group.

**FIGURE 7:**
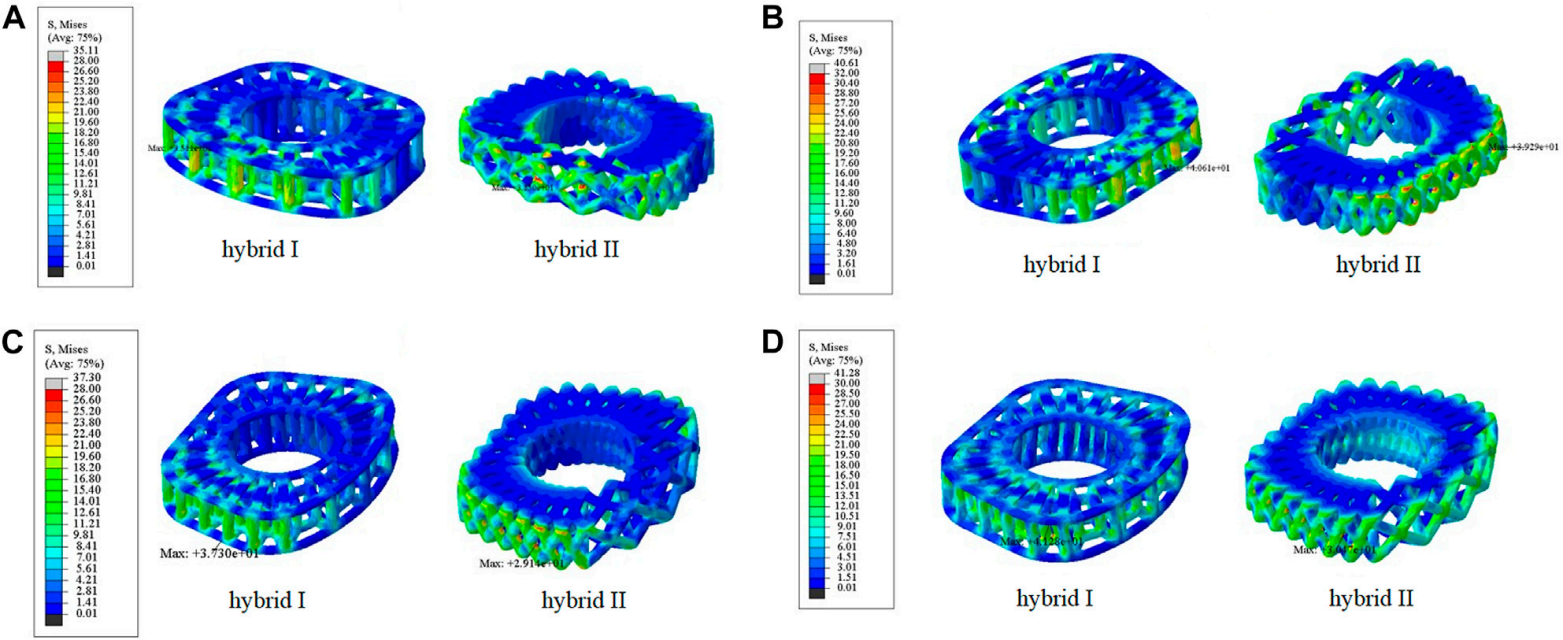
Von Mises stress distributions on the lattice fiber jacket for the hybrid I and hybrid II groups for different motions: **(A)** flexion, **(B)** extension, **(C)** lateral bending, and **(D)** axial rotation. (unit: MPa).

According to the comparison of the biomechanical performance of the intact model for the range of motion under various loadings, the optimal lattice structure distributions from the Hybrid I and Hybrid II groups were A2L5P2 in the Hybrid I groups and A2L7P3 in the Hybrid II group, and they were selected to be compared with the Baguera^®^C group (Figure 6). All the maximum von Mises stresses of the PCU fiber jacket were within the yield strength of the PCU material (less than 41.1 MPa) (Inyang and Vaughan, 2020; Ford et al., 2018) except for one value of 41.28 MPa in the Hybrid I group under axial rotation (Figure 7).

### Range of motion results for the two optimized hybrid groups, Baguera^®^C group and intact group

**FIGURE 8:**
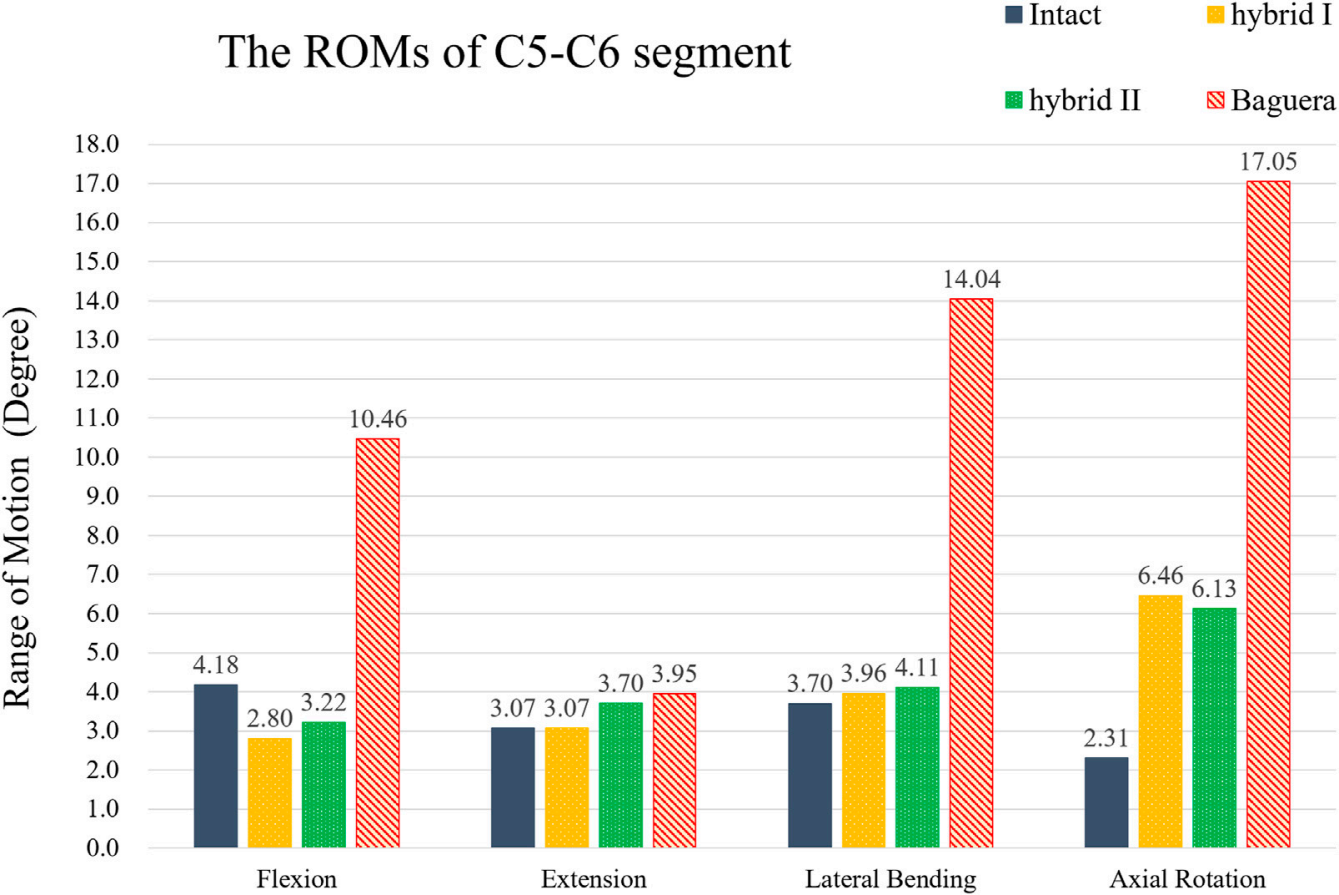
ROMs of the C5-C6 segment of hybrid I, hybrid II and Baguera^®^C groups compared to those of the intact group.

To compare the primary stabilizing properties of the three TDR groups, the ROMs of the operated C5-C6 motion segments were compared. The ROMs of the C5-C6 models of the Hybrid I, Hybrid II, and Baguera groups were compared with those of the intact group under the follower load of 100 N and pure moment of 1.5 Nm, and the ROMs 33% less, 23% less, and 150.2% more in flexion; unchanged (0%), 20.5% more, and 28.7% more in extension; 7.0% more, 11.1% more, and 279.5% more in lateral bending; and 179.7% more, 165.4% more, and 638.1% more in axial rotation, respectively (Figure 8).

### Stress analysis among the two optimized hybrid groups, Baguera^®^C group, and intact group

#### Core stress

**FIGURE 9:**
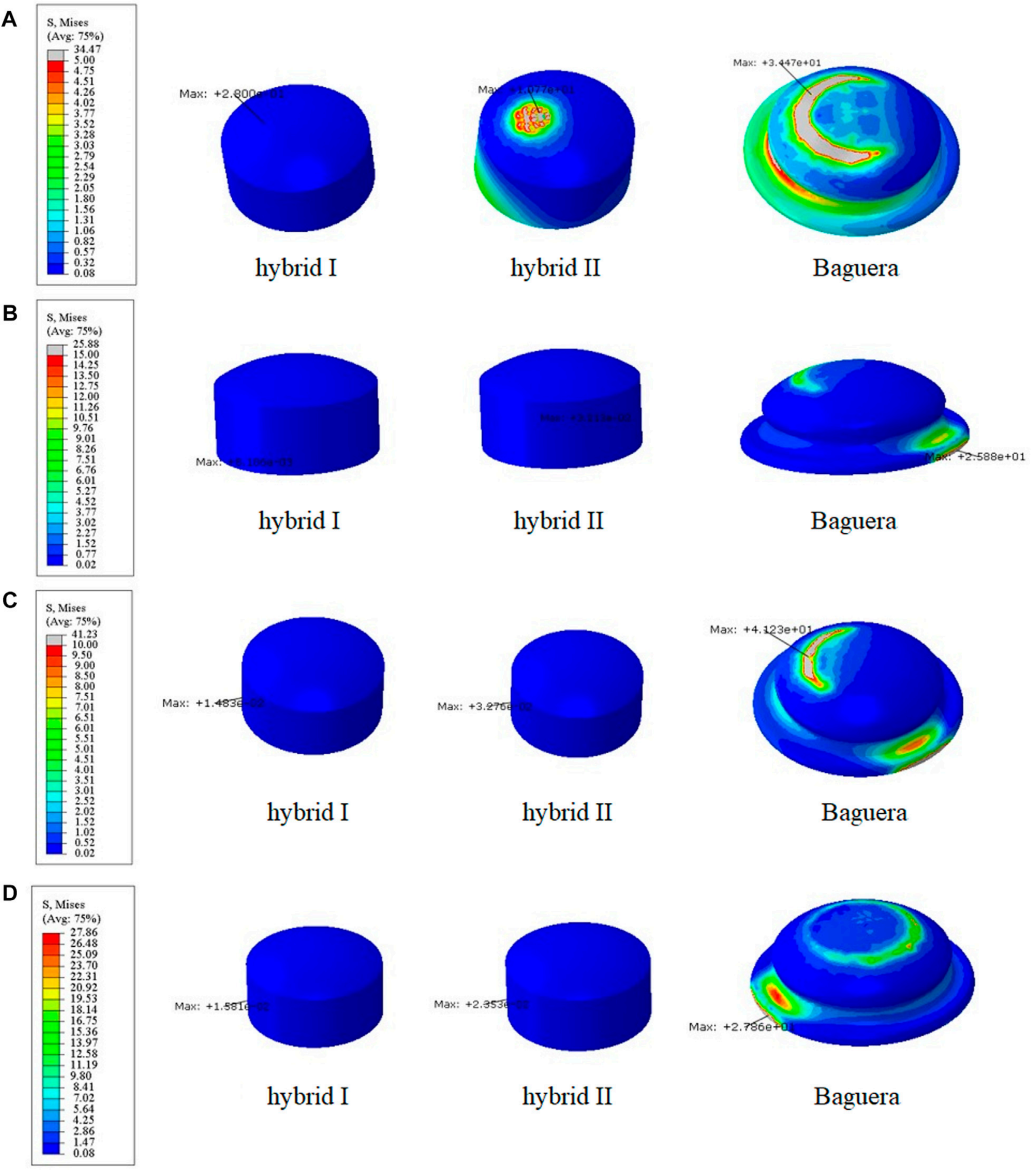
Core stress distribution patterns for the Hybrid I, Hybrid II, and Baguera groups for different motions: **(A)** flexion, **(B)** extension, **(C)** lateral bending, and **(D)** axial rotation. (unit: MPa).

To evaluate the durability of the prostheses, the von Mises stress distributions of the cores during four different motions were analyzed (Figure 9). In flexion, the stress distribution in the Hybrid I group was the lowest (0.28 MPa in the Hybrid I group, 10.77 MPa in the Hybrid II group and 34.47 MPa in the Baguera^®^C group). In extension, lateral bending and axial rotation, both the Hybrid I and Hybrid II groups showed considerably lower stress values (0.01 MPa, 0.01 MPa and 0.02 MPa in the Hybrid I group; 0.03 MPa, 0.03 MPa and 0.02 MPa in the Hybrid II group) than the Baguera group (25.88 MPa, 41.23 MPa, and 27.86 MPa, respectively).

#### C6 vertebral superior endplate stress

**FIGURE 10:**
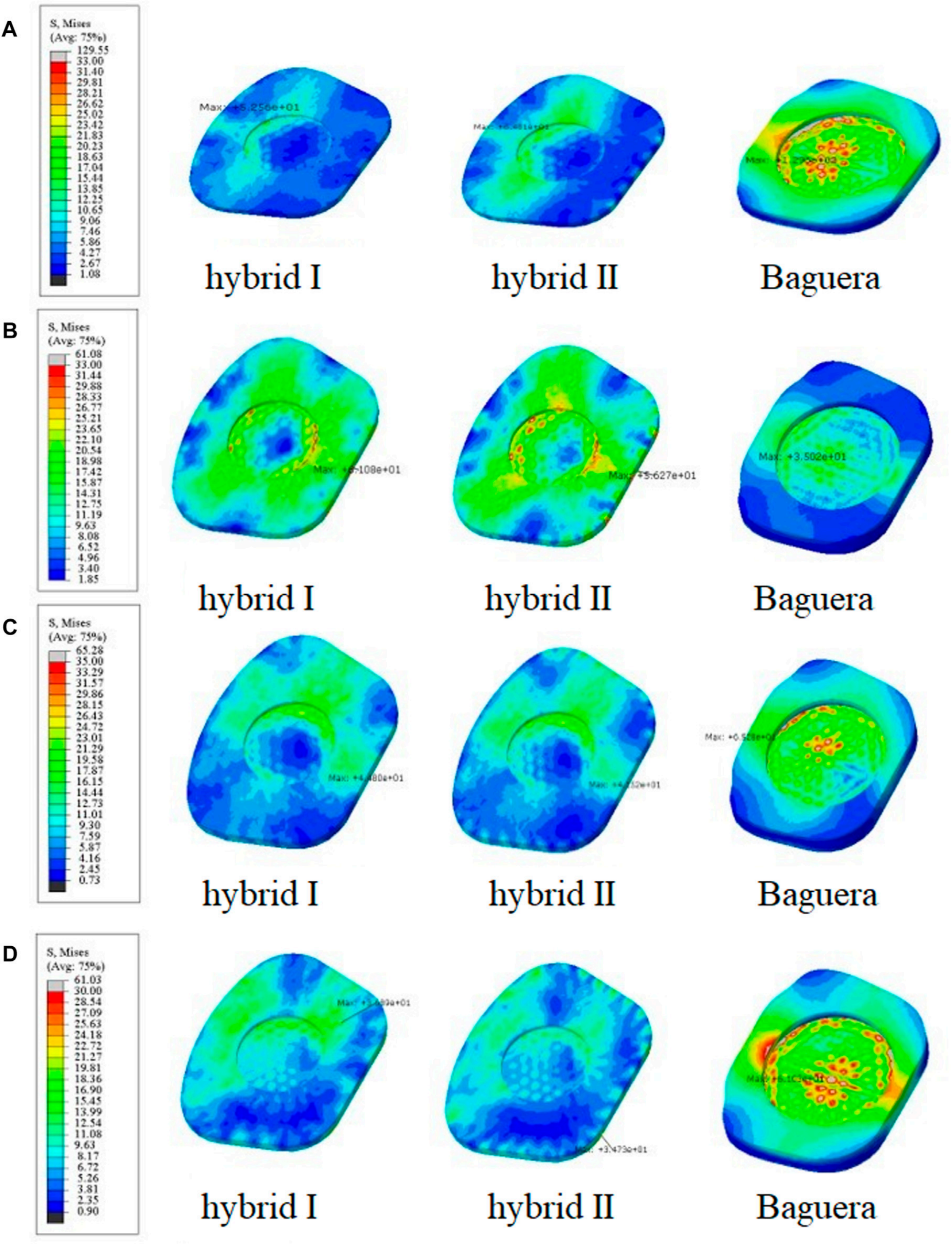
Von Mises stress distributions on the C6 superior endplate for the hybrid I, hybrid II and Baguera^®^C groups for different motions: **(A)** flexion, **(B)** extension, **(C)** lateral bending, and **(D)** axial rotation. (unit: MPa).

To predict the tendency of prosthesis subsidence, the von Mises stress distribution on the interface between the prosthesis and C6 vertebral superior end plate was evaluated (Figure 10). In flexion, the stress values were 52.56 MPa in the Hybrid I group, 64.01 MPa in the Hybrid II group and 129.6 MPa in the Baguera group. In extension, the results were 61.08 MPa in the Hybrid I group, 56.27 MPa in the Hybrid II group and 35.02 MPa in the Baguera^®^C group. In lateral bending, the results were 44.80 MPa in the Hybrid I group, 41.52 MPa in the Hybrid II group and 65.28 MPa for the Baguera group. In axial rotation, the results were 36.89 MPa for the Hybrid I group, 34.73 MPa for the Hybrid II group and 61.03 MPa for the Baguera^®^C group. Overall, the von Mises stress level on the C6 superior endplate was much greater in the Baguera group than in the Hybrid I and Hybrid II groups, as shown in Figure 10. In flexion, extension and axial rotation, a larger stress concentration on the margin of the Baguera group was observed, which indicated a greater risk of subsidence.

#### Facet stress

**FIGURE 11:**
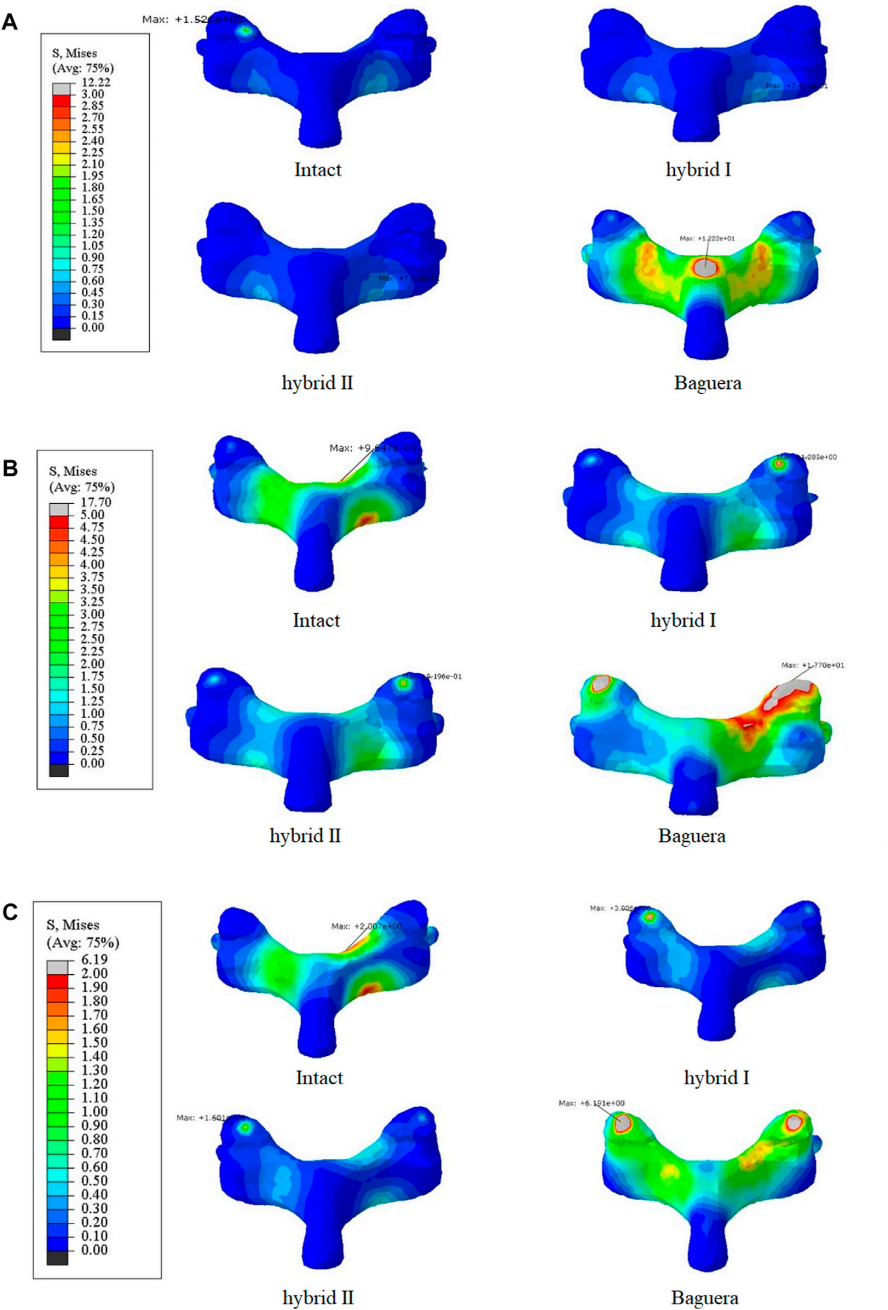
Von Mises stress distributions on the facet joints for the intact, hybrid I, hybrid II and Baguera^®^C groups for three different motions: **(A)** extension, **(B)** lateral bending, and **(C)** axial rotation. (unit: MPa).

The facet stress distributions of the four groups during three different motions (extension, lateral bending and axial rotation) are shown in Figure 11. In extension, the stress values were 1.53 MPa in the intact group, 0.0 MPa in the Hybrid I group, 0.0 MPa in the Hybrid II group and 4.03 MPa in the Baguera group. In lateral bending, 0.16 MPa in the intact group, 1.29 MPa in the Hybrid I group, 0.92 MPa in the Hybrid II group and 17.70 MPa in the Baguera group. In axial rotation, 0.10 MPa in the intact group, 3.01 MPa in the Hybrid I group, 1.60 MPa in the Hybrid II group and 6.19 MPa in the Baguera group. Both the Hybrid I and Hybrid II groups showed relatively lower facet stress than the Baguera group; furthermore, facet stress values of the Hybrid II group showed a similar tendency to the intact group than the Hybrid I group. Among the three different motions in the Baguera group, significantly increased facet stress was observed in lateral bending, which indicated that lateral constraint of the prosthesis was lacking.

### Paths of ICRs among the two optimized hybrid groups, Baguera^®^C group, and intact group

**FIGURE 12:**
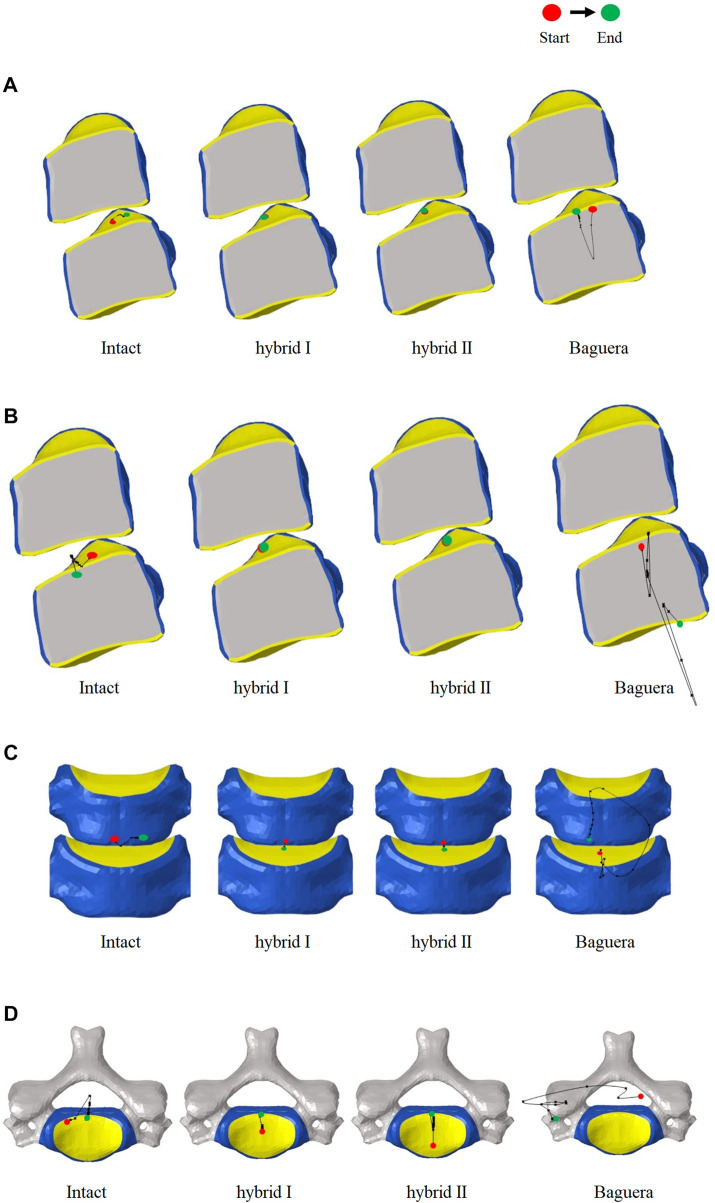
Paths of ICR for the intact, hybrid I, hybrid II and Baguera^®^C groups for different motions: **(A)** flexion, **(B)** extension, **(C)** lateral bending, and **(D)** axial rotation.

The path of the ICR, an alternative of ROM, has been proposed to evaluate the quality of spine movement and to identify abnormal cervical spine kinematics (Kamal and Rouhi, 2016b; Kim et al., 2019). The detailed ICR paths were calculated through the relative positions of the vertebrae during motion as described in the literature. During C5-C6 treated level movement, a total of 20 ICRs from each individual segment were calculated to form the path of ICRs. In flexion, extension and lateral bending, the ICR paths of the Hybrid I and Hybrid II groups did not change much, whereas the ICR path of the intact group shifted sagittally in flexion/extension and shifted coronally in lateral bending (Figure 12). In axial rotation, the ICR paths of the intact and hybrid groups shifted posteriorly, which was in agreement with other studies (Muhlbauer et al., 2022; Venegas et al., 2020; Claessens, 2017). In the Baguera^®^C group, obvious anterior shift of the ICR in flexion, posterior-inferior shift in extension, and deviated and irregular movements in lateral bending and axial rotation were recorded.

## Discussion

As observed in the literature, total disc replacement at the C5–C6 segment with a non-articulating prosthesis, such as the M6 cervical disk prosthesis (Spinal Kinetics, Sunnyvale, CA, United States) with PCU core and PE fiber to mimic normal disc anatomy, has been proven clinically to be a superior alternative because of the benefits of maintaining ROM and smaller heterotopic ossification rate (Patwardhan et al., 2012; Phillips et al., 2021; Oltulu et al., 2019). The kinematic response and postoperative flexion/extension stiffness were proven to be biomechanically compatible with the intact motion segment. However, significantly greater stiffnesses in lateral bending and axial rotation were still obstacles (Patwardhan et al., 2012). Due to the load-bearing capabilities and complexity of motion exposure to daily activities, the importance of evaluating the material characteristics of the prosthesis intended to replace the disc cannot be underestimated. Furthermore, current failures associated with existing implants, such as heterotopic ossification, insufficient stiffness, dislocation, wear and improper ICR further buttress the need for assessing the mechanical behavior of a material proposed to replace the disc (Virk et al., 2021; Shen et al., 2021; Shen et al., 2022; Cao et al., 1976; Parish et al., 2020). Thus, an appropriate material attribute design using a PE core and PCU fiber jacket with adequate native geometry could represent a suitable candidate for replacing the disc. polycarbonate urethane (PCU) could be customized and adapted based on a suitable choice of reinforcement fiber, and the property of fiber-reinforced composite enabled the load-bearing nucleus from dislocation and have been applied in joint replacement (Inyang and Vaughan, 2020; Ford et al., 2018). Biomechanical properties such as modulus of elasticity compatible to the annulus fibrosus make PCU a more suitable surrounding fiber candidate than PE.

Lattice structures have become a key factor for AM prostheses since AM processes enable the fabrication of cellular materials with complex microstructures and mimic disc spatial biomechanical behavior (dellaRipa et al., 2021; Pan et al., 2020; Chen et al., 2017). Several lattice cell structures could be used in this study, including X, Star, Tesseract, Vintiles, Cross, and Octet. However, due to the potential for implant instability, lattice structures that exhibited stress over-concentration at the implant endplate in X and Vintiles, as well as a micro-buckling effect under compressive loads in Octet, were excluded. Ultimately, the Tesseract (Hybrid I group) and Cross (Hybrid II group) structures were selected for analysis due to their stable and nonlinear mechanical properties, as well as their ability to mimic the biomechanical behavior of natural discs (Weeger et al., 2019). Because the material properties of the annulus fibrosus were varied linearly in the circumferential and radial directions histologically (Zhu et al., 2008), the circumferential area of the PCU fiber jacket was divided into three different regions, and the cellular density was adjusted. According to the biomechanical performance in the range of motion under various loadings compared to the intact disc, the optimal cellular densities were A2L5P2 from the Hybrid I group and A2L7P3 from the Hybrid II group. From the values and distributions of von Mises stress of both chosen Hybrid groups, the Hybrid II group showed more symmetric and lower stress than the Hybrid I group. The Tesseract structure in the Hybrid I group was the four-dimensional analog of a cube and lacked the ability to resist torsion due to vertical alignment, whereas the Cross structure in the Hybrid II group absorbed direct force and efficiently buffered the impact. In Figure 6, a Cross structure with smaller size than the Tesseract structure was made by the innate programed lattice structure, which induced greater cellular density in the Hybrid II group than the Hybrid I group and thus, more even stress distribution was found in the Hybrid II group.

The main purpose of this finite element study was to investigate the biomechanical performance of the newly designed additively manufactured hybrid TDR and compare it to the performance of the Baguera^®^C prosthesis with downward mobile core under loadings that simulated the daily activities of patients by imposing a pure moment of 1.5 Nm combined with a follower load of 100 N. Our data showed a difference in the lateral bending and axial rotation at C5/C6 compared to a previous *in vivo* study using ten young asymptomatic adults, which reported values of 6.11 and 3.01°, respectively (Lin et al., 2014). It should be noted that the *in vivo* study measured dynamic cervical intervertebral ROMs during active motion in the sitting position, while our study imposed a pure moment and a follower load passively. However, the ROMs of our intact C5-C6 FE model were comparable to previously obtained experimental data using the same moment and follower load (Finn et al., 2009; Purushothaman et al., 2020).

From the results for each implant group, both hybrid groups underwent similar ROMs and had core stress distributions similar to that of the intact group in contrast to the Baguera^®^C group (Figures 8, 9). The deformation property of the intervertebral disc allowed flexibility under smaller loadings and restricted excessive motion under larger loadings. TDRs with ball-and-socket designs can theoretically provide benefits as movable articulating prostheses, but facts have shown that moments cannot be constrained in flexion, lateral bending or axial rotation. The Baguera^®^C disc prosthesis had been proven to have stiffness similar to that of the intact disc and mobile core design in contrast to prostheses with other ball-and-socket designs (Kamal and Rouhi, 2016a), but they still showed obviously higher ROM values than our two hybrid groups.

One of the major complications of current commercial TDR designs is subsidence resulting from interfacial stress concentration on the bony endplate. Anchorage of ball-and-socket TDR using central fins or teeth might cause higher stress concentrations, thus initiating stress shielding and bone resorption (Lin et al., 1976). Another reason for subsidence is hypermobility, which causes excessive strain over the facet joint and surrounding capsular ligaments and indirectly increases stress over the anchorage area between the prosthesis and bone (Kerferd et al., 2017; Matgé et al., 2015). The C6 vertebral superior endplate stress was focused unevenly and mostly on the central regions in the Baguera group but was evenly distributed in the peripheral region of both hybrid groups in all ROMs (Figure 10). The top and bottom boundaries of both hybrid groups were chosen to conform evenly to the vertebral endplates with stainless steel. The nonlinear behavior of the lattice structure arrangement in the PCU fiber jacket can resist compression, constrain expansion and dissipate the concentrated load.

In extension, lateral bending and axial rotation, both the hybrid I and hybrid II groups showed facet stress distributions similar to that of the intact group (Figure 11). In the Baguera^®^C group, stress focused on the lamina in extension, unilateral facet in lateral bending and both facets in axial rotation due to the lack of a constraint of the artificial annulus fibrosus, which was compatible with other finite element studies (Rong et al., 1976). Increased facet joint stress after ball-and-socket TDR has been discussed as a reason for degenerative changes at the surgical level and leads to poor clinical results (Rong et al., 1976; Rousseau et al., 1976; Wang et al., 2021). Rousseau et al. showed an increase in facet stress from 18% to 86% with increasing load from 0.8 to 1.6 Nm in a FE model and recommended posterior implantation of a larger radius prosthesis to diminish the elevated stress (Rousseau et al., 1976). Gandhi et al. compared two TDRs (Bryan and Prestige LP) in the C2-T1 reconstructed finite element model and found that the facet contact force increased by 50%–100% compared with the intact disc in the C5-6 operated level (Rong et al., 1976). Wang et al. measured the facet pressure in six C5-C6 cadaveric spines implanted with different sizes of ball-and- socket TDRs and found significantly elevated facet joint pressures from 25.2 to 44.6 psi in flexion and from 58.9 to 90.3 psi with a 2 mm increment in prosthesis height (Wang et al., 2021). Improper positioning or size of the device and intrinsic design defects might have been the reasons for elevated stress. Elastomers including a polymer fiber jacket resulted in nonlinear behavior similar to that of an intact disc (van denBroek et al., 2012). This nonlinearity provides a neutral zone to protect surrounding tissues from high loading.

The ICR path has been proposed as an alternative to the ROM for evaluating the quality of spine movement and for identifying abnormal cervical spine kinematics. In normal discs, the average location of the ICR path was observed to be posterior to the geometric center of the inferior vertebral body and move anterior-posteriorly during flexion/extension (Anderst et al., 1976). The ICR location was not significantly affected superior-inferiorly during motion. In our study, the ICR paths of the Hybrid I and Hybrid II groups were similar to that of the intact group in all ROMs; however, in the Baguera group, an obvious anterior shift of the ICR in flexion, posterior-inferior shift in extension, and deviated and irregular movement of lateral bending and axial rotation were also observed. A systematic review showed that the ICR of ball-and-socket TDR tended to shift anteriorly or superiorly in finite element studies or *in vitro* biomechanical studies and may have been easily affected by the prosthesis implantation position (Patwardhan et al., 2012; Sang et al., 2020). The Baguera^®^C group, with a two-piece articulation design, constrained the ICR to the center of the radius of curvature of the prosthesis, and a downward center of rotation of Baguera^®^C caused ROMs to be more similar to the intact model than other implant design (Kowalczyk et al., 2011; Kamal and Rouhi, 2016a). In studies using ProDisc-C^®^ (Synthes, West Chester, PA, United States), the ball on the lower endplate (possessing the same radius of curvature as the socket on the upper plate) provided an ICR at a more inferior location at the ROM. Studies comparing the sagittal kinematics of postoperative ICRs above the midline of the disc space with an upper-located ball design, Prestige LP^®^, and a lower-located design, ProDisc-C^®^, suggested that the location of postoperative ICR was strongly correlated with the artificial disc design, and neither design could not fully replicate the physiological ICR path (Rong et al., 1976).

In this study, we aimed to determine which additively manufactured hybrid group was a cervical joint prostheses that mimicked an intact disc and compared it to a commercial product for further clinical recommendation. There were some limitations in our study. First, certain assumptions were made in this parametrized finite element model, and an *in vivo* animal study should be performed for validation in the future. Second, more commercially available cervical joint prostheses, including one with a lower ball or a six-degree prosthesis with a PCU core and PE fiber design, are still needed to make a comprehensive comparison. Third, partial ALL or PLL should be removed to reveal the clinical operative segment. Finally, a longer cervical segment could be created for further evaluation of adjacent segment stress and degeneration.

## Conclusion

Ranges of motion and core stresses similar to those of the intact group indicate the possible longevity of the proposed additively manufactured hybrid TDR. Lower endplate stress and facet force compared to those of the Baguera^®^C group offer a solution for preventing implant subsidence. The ICR path of the additively manufactured hybrid groups in all ROMs were similar to those of the intact disc and can restore normal cervical spine kinematics. Superior stress distribution of the PCU fiber jacket and core in the additively manufactured hybrid II group compared to the Hybrid I group revealed that the Cross lattice structure of the PCU fiber jacket could be a choice for a next-generation TDR. In summary, this promising outcome suggests the feasibility of implanting an additively manufactured multi-material artificial disc that allows for better physiological motion than the current ball-and-socket design.

## Data Availability

The original contributions presented in the study are included in the article/Supplementary Material, further inquiries can be directed to the corresponding author.
