# Supplementary material for: Finite element analysis of optimized novel additively manufactured non-articulating prostheses for cervical total disc replacement
Source: Front Bioeng Biotechnol. 2023 Jun 1;11:1182265. doi: 10.3389/fbioe.2023.1182265 (PMC10267663; doi:10.3389/fbioe.2023.1182265)
Supplement: Supplementary file 1 [file Table1.docx]

Supplementary 1. Finite element model convergence test

| Intact | | | |
| --- | --- | --- | --- |
| convergence mesh size | Number of mesh | Total strain energy (J) | error (%) |
| 1.5 | 90,804 | 58.1829 | 0.70% |
| 1.4 | 95,770 | 57.8009 | 0.04% |
| **1.3** | **107,607** | **56.9155** | **1.49%** |
| 1.2 | 134,590 | 58.2706 | 0.86% |
| 1.1 | 183,284 | 57.7765 | 0 |
| hybrid I | | | |
| convergence mesh size | Number of mesh | Total strain energy of artificial annulus fibrosus (J) | error (%) |
| 0.3 | 365,262 | 24.8311 | -4.55% |
| 0.25 | 399,318 | 25.1552 | -3.30% |
| **0.2** | **539,896** | **25.4135** | **-2.31%** |
| 0.15 | 792,950 | 25.7212 | -1.12% |
| 0.1 | 1,349,050 | 26.0137 | 0.00% |
| hybrid II | | | |
| convergence mesh size | Number of mesh | Total strain energy of artificial annulus fibrosus (J) | error (%) |
| 0.3 | 293,440 | 28.2021 | -2.88% |
| 0.25 | 360,294 | 28.5774 | -1.59% |
| 0.2 | 480,922 | 28.6662 | -1.28% |
| **0.15** | **777,046** | **29.0656** | **0.09%** |
| 0.1 | 1,365,754 | 29.0386 | 0.00% |
| Baguera | | | |
| convergence mesh size | Number of mesh | Total strain energy of artificial disc core(J) | error (%) |
| 0.3 | 173,073 | 2.0359 | 10.83% |
| 0.25 | 265,228 | 2.1202 | 7.14% |
| **0.2** | **343,241** | **2.2295** | **2.35%** |
| 0.15 | 610,634 | 2.3017 | 0.81% |
| 0.1 | 1,368,193 | 2.2832 | 0.00% |
